# What’s on the menu? A qualitative study on the views of care home staff and residents on nutritional practices and implications for oral health

**DOI:** 10.1038/s41405-025-00325-9

**Published:** 2025-04-01

**Authors:** Aditi Mondkar, Maritess Murdoch, Jennifer E. Gallagher, Rakhee Patel

**Affiliations:** 1 Consultant in Dental Public Health, NHS England South East, Wellington House, 133-155 Waterloo Road, London, SE1 8UG UK; 2 Service Development, Integration, Transformation and Delivery Lead (Age Well), NHS North Central London Integrated Care Board, Laycock Street, London, N1 1TH UK; 3 Newland-Pedley Professor of Oral Health Strategy/ Honorary Consultant in Dental Public Health, Centre for Host Microbiome Interactions, Faculty of Dentistry, Oral & Craniofacial Sciences, King’s College London, Denmark Hill Campus. Bessemer Road, London, SE5 9RS UK; 4 Senior Clinical Lecturer and Honorary Consultant in Dental Public Health, Centre for Host Microbiome Interactions, Faculty of Dentistry, Oral & Craniofacial Sciences, King’s College London, Denmark Hill Campus. Bessemer Road, London, SE5 9RS UK

**Keywords:** Nutrition and diet in dentistry, Oral hygiene

## Abstract

**Introduction:**

People living in nursing or residential homes are at an increased risk of having or developing oral and dental diseases. This is due to contributing factors such as comorbidities and polypharmacy coupled with poor manual dexterity and lack of disease prevention and access to care. These risk factors combined with frequent and high sugar consumption increases risk of developing dental decay. Care home residents are a diverse population from different backgrounds. Little is known about decision making around nutrition in care settings and nutritional practices of older people in care homes, including the choices and challenges. The purpose of this study was therefore, to understand care home landscape and resident experiences.

**Materials and methods:**

Semi-structured interviews were conducted with participants including residents, care home managers, carers and kitchen staff. Interviews were held face-to-face, recorded and transcribed and the findings were analysed using a thematic approach.

**Results:**

A total of 17 participants across 4 care homes in one outer London borough took part in this study, with findings relating to both nutrition and oral health. Resident food preferences were collected upon admission into the home, including information on the incoming resident’s lifestyle, routine and choices. Staff tried to maintain these where possible and accommodate to resident choices when possible. Opportunities for wider food choice was dependent on the residents’ ability and willingness to request alternatives, their financial freedom to purchase their own foods, mobility to access food outside of the care home setting and food bought in by family and friends. This was compounded by residents being prescribed high calorie, high sugar meal supplemented for weight management. Participants reported that the structured routine revolved around meals and activities. Most care staff reported that an alternative healthy option was offered at mealtimes, but this was not the case in all homes. Care staff faced challenges managing weight of residents who had appetite loss and tried to accommodate and make provisions for those with these experiences and were aware of how to raise concerns. There was variation in mandatory training expectations, with no integration of oral health and nutrition.

**Discussion:**

The findings provided valuable insight into the disparities between and within homes and highlights the challenges in this complex group with regards to nutritional choices. It is vital that a range of food options are in place to protect residents’ rights to choose what they wish to eat, whilst offering healthy options and all care staff are educated on offering balanced, nutritious meal and snack options. By offering a range of foods, the healthier choice can be the easier choice.

## Background

Globally the population is living longer, with the World Health Organisation (WHO) projecting the population of those aged 60 and older set to increase from 12 to 22% between 2015 to 2050 [1].

Although we are living longer, we are not living better. In the United Kingdom (UK) over the last decade, disability free life expectancy (DFLE) has increased, but not at the same rate at life expectancy [2]. There are inequalities in life expectancy and disability with deprived communities carrying the burden disease [2]. As people enter their later stage of life, their level of dependency increases and they are often cared for by informal or formal carers either in a their own home or a care home setting [2]. In 2024, approximately 441,479 people were estimated to be living in care homes in the UK with varying degrees of frailty and dependent care needs [3].

Over the past few years, oral health in care homes has become an increasingly important topic for consideration. Older people are more likely to have poor oral health and less likely to access dental services [4–7]. Difficulty in eating can result in inadequate intake of required nutrition, impact on wellbeing and is associated with poorer general health [6–9]. Evidence shows older adults admitted into care homes are at greater risk of becoming malnourished [10–13]. Furthermore, older adults are more likely to be affected by multiple comorbidities, side effects from medications and poorer manual dexterity, limiting self-care and their ability to undertake oral health practices.

As people age, their food choices are often dictated by their ability to masticate and maintain oral function [14–16]. When people lose weight, frequent intake of high calorie foods are often high in fat and high in sugar, such as high calories replacement meals [17]. There is clear benefit in care settings observing good, healthy nutrition practices in providing an environment that supports a stronger emotional and physical wellbeing, in addition, food is a memorable experience.

Excessive and frequent sugar intake amongst all ages is a known common risk factor for several health conditions, one of which is dental decay [18]. A review by Kassebaum et al., (2017) estimated global prevalence and incidence of oral conditions (i.e., untreated dental caries, periodontitis, edentulism, and other oral disorders) of older adults at 57% and 77% respectively [19]. As people age, they are retaining their teeth for longer. In the 2009 UK Adult Dental Health Survey (ADHS) 53% of those aged over 85 were dentate, and they retained on average 14 teeth [20]. Increased frequency and quantity of non-intrinsic/free/added sugars increases the risk of dental decay [21]. The 2016 Public Health England review of oral health of older people in England and Wales reported higher prevalence of dental caries [22–24] in care home residents than their community dwelling peers [20].

Guidelines produced by the UK National Institute of Clinical Excellence (NICE) in 2016 highlighted the need for timely access to dental treatment and policies on the oral health, and use of the oral health assessment tool and mouth care plans in the care home; including equipping care staff with the knowledge and skills to support residents [25]. The Care Quality Commission (CQC) reviewed oral health in care homes in 2019 (updated 2023) and the results showed that oral health is still not a priority in care homes and many residents were unsupported with maintaining their oral hygiene [26, 27].

Teeth are being retained across a lifetime and so it is vital that measures are put into place to prevent oral health diseases and maintain good oral hygiene throughout the lifecourse. Central to this is maintenance of good oral health and dietary practices wherever possible.

However, there is a paucity of information around the decision making around nutrition in care settings, including the choices and motivations for high sugar intake and whether this is care home or resident motivated. Insights into dietary practices could support care homes to deliver nutritionally balanced food items that concurrently mitigate the risk of developing dental decay and other sugar related general health conditions.

The aim of this study was to understand the care home landscape and resident experience in relation to nutritional and oral health practices. These were addressed through the following objectives:

- To understand local policy arrangements in relation to nutritional practices and oral health in care homes
- To assess oral health and nutrition practices from stakeholders’ perspective including care home teams (managers, chef, carers) and residents
- To explore residents’ experiences in oral health and nutrition.

## Methods

### Sampling

This study was carried out as part of a service review undertaken jointly by the public health and commissioning teams in one London borough, utilising qualitative methodology. Four of ten homes in the borough were purposively sampled and invited to take part in this study.

Only homes registered to care for people aged 65 and older (with and without dementia care) were included in the study. Local authority and commissioning colleagues familiar with the landscape advised on homes that were to be excluded based on wider challenges, such as those in special measures that would not be suitable to participate. Care homes were the stratified by size- small (0–40), medium (40–65), large (65+ beds), and type of care (nursing or residential).

In acknowledgement of the challenges of engaging with social care and care homes, a working group was established consisting of key stakeholders including representatives from Public Health England Dental Public Health, Local Authority and the Clinical Commissioning Group. Contact with representatives from the Care Homes forum and nutritional team were also invited as needed. The purpose of this group was to shape the approach and engage as a collaborative with care homes, as well as to feedback to the sector on findings that could inform local level changes.

Once homes were identified and recruited, care home participants, including care home managers, health care assistants, kitchen staff and residents were invited to undertake qualitative interviews with a trained researcher. As the researcher recruiting participants was also the person collecting the qualitative data, care was taken to not introduce moderator bias. Participants were unaware of the researcher’s dental background to avoid responder bias. Iterative reflective practice was undertaken by the researcher in acknowledgement of potential relationship imbalances and reflect honestly and openly on its potential effect on study findings throughout the process.

Only English speaking and residents with capacity were invited to take part in the study. Suitable residents were identified by the care home mangers. If the researcher held concerns about capacity, interviews ceased. Any incomplete interview data was not included in the analysis. Written consent was obtained on the day, and interviews were recorded and transcribed verbatim.

A topic guide was developed and explored topics such as structure of the home, planning of meals, training and challenges associated with nutrition and oral health. Interviews were undertaken face to face at the care home and were arranged at a time convenient to the participant(s) and took between 30-40 minutes.

### Data analysis

Thematic analysis via a matrix-based approach to qualitative data management and analysis was applied. Data analysis was an iterative process, with transcripts being re-read and reviewed throughout data collection. A framework based on the literature review and topic guide, was applied to the data with emergent themes added. Data was then indexed, sorted, and reviewed according to relevant themes and sub themes by hand and using Microsoft Excel. The data were summarised and categorised across the transcript and descriptive explanations of the themes developed [28].

### Ethical approval

This study was approved by the Public Health England Research Ethics and Governance Group (Ref: NR0156).

## Results

Four care homes agreed to participate in this study, with size of homes ranging from 36 to 117 beds. Three homes were residential care homes, and one was a nursing home. A total 17 participants were interviewed in 2019; there was a balance of participants in each home, with the following roles: four (24%) care home managers, five (29%) healthcare assistants, four (24%) kitchen staff and four (24%) residents. There were more females (*n* = 10, 59%) than males (*n* = 7, 41%) participating. Table 1 presents the demographics of the care homes and participants.

**Table 1:** Participant demographics

Care Home	Care Home Type	Number of beds	Participant Type								Total Number of Participants
			Care Home Manager		Healthcare Assistant		Kitchen Staff		Resident		
			Participant number	Gender	Participant Number	Gender	Participant Number	Gender	Participant Number	Gender	
**1**	Nursing	117	P13	Male	P15	Female	P17	Male	P3	Female	**4 (24%)**
**2**	Residential	36	P14	Male	P11	Female	P5	Female	P4	Male	**4 (24%)**
**3**	Residential	40	P7	Female	P8	Female	P6	Female	P1	Male	**4 (24%)**
**4**	Residential	62	P12	Male	P2	Female	P10	Male	P9	Female	**5 (29%)**
					P16	Female					
	**Total Participants by Type**		**4 (24%)**		**5 (29%)**		**4 (24%)**		**4 (24%)**		**17**
								**Total Participants by Gender**			
									**Male**	**Female**	
									**7 (41%)**	**10 (59%)**	

The combined participant themes related to both nutrition and oral health and included, resident food preferences, eating and drinking between meals, menu development, weight management and staff training.

### Resident food preferences

On admission, all participating care homes collected information about the incoming residents from residents themselves or family members; this included diet preferences to ensure that they were catered for. Daily activities were structured around mealtimes and food formed part of the activities. Some homes recognised the opportunity of mealtimes to maintain the senses such as taste and smell, as well as the social aspect that comes with eating meals together giving residents the opportunity to talk and interact with each other.

*“Food is a wonderful way as you would do at home…to structure the day. So, for us the dining experience…the experience of having food and eating is an activity in itself. And actually, one of the last few sensual pleasures left to older people who are perhaps in a decreasing mobility and their hearing and their vision. You know some of them, if there’s a good sense of smell then they can taste quite well, still, and then a good meal is a source of peace and joy.”* Participant 7, Staff member

Residents were provided menus in advance to choose their meal choices for the next day. Some residents had family members involved in deciding their food choices. Residents had a choice of where they wanted to sit to eat, for example in the dining area or in their room,

*“…they have choice for the next day every day, for the next day, mainly like at lunch and dinner…it’s like a piece of paper, so when they were admitted they ticked the boxes what are their preferences for breakfast, whether its brown bread, whether its porridge or whether its cornflakes for breakfast… lunch and dinner they can pick up their own choices every day, so they know at lunch what they are having.”* Participant 2, Staff member

*“We just show them…and they will choose. Because if …sometimes that they are not able to communicate, but they are able to you know, their facial expression or their body language will tell you that oh this is what I really want, and sometimes to their relatives also give us this information…oh my mom likes this, oh my mom likes that, oh my dad likes this, or that is where we work alongside with the relatives to get information as well.”* Participant 15, Staff member

Flexibility to the menu added an element of choice to residents. Most care home staff reported to be accommodating to the residents wishes and if an option was not suitable for a resident, alternative arrangements were prepared, or decisions were made with family members for options. However, this varied home to home.

*“Yes, they would do that for you specially* *[if wanted to eat something in particular]* *… sometimes like today there was some foods that I don’t like so I asked if I could have a salad please and they will do that for me.”* Participant 3, Resident

*“…we do have a menu that goes in for the whole month and it is all planned, and everybody knows what is on the menu but if at any point on the day if a resident says that they do not wish to have any other food and they want something else, that is done. So therefore, it is not a regimented that whatever is provided on the menu is all that they will have, but no, they have their choice…”* Participant 13, Staff member

Staff were aware that flexibility encouraged residents to be eating well. The carers looked at what was being consumed as an indication of how well the residents were that day, and modifications were made to support resident’s needs.

*“….they have an offer like lunch and evening time, when it is the dessert time, you can choose between ice cream, because its soft, some people like soft, you can choose between fruit salad or cheese and biscuits or jelly, yes, so they have a choice, if someone has a problem with the swallowing, we try to blend it and mix it…”* Participant 2, Staff member

In some cases where there was lack of flexibility with no suitable menu option, residents who were able to would go out to purchase the food items which they enjoyed.

*“It’s just whatever they cook you, basically. It’s very rare I deviate, just accept whatever they’re serving. You know, you don’t get a choice… well you get offered something like say, you get offered barbecue chicken or broccoli quiche. You get two options…I never ask for anything* *[if the option for lunch as not liked]…I would much rather, like today I will go out and eat because I don’t like what’s being served today, I will go out and eat. And pay for it myself.”* Participant 4, Resident

### Eating and drinking between meals

All residents in the participating care homes had access to ‘hydration stations’ which had water and juices available. Residents were also offered or could ask for tea/coffee which were prepared by carers. There was variation in snacks provided between meals, but mainly reported as biscuits and cakes. Some staff reported that residents had access to fruit baskets in the kitchenette or residents were able to ask staff for fruit. Not all homes offered fruit as a snack. Some residents purchased fruits for themselves, or they ate them as part of dessert during main meals.

*“You’re not given fruit separately, it’s part of your meal. Like rather than have yogurt, they may give you a banana.”* Participant 4, Resident

*“Snacks, it’s usually biscuits, but if they request fruit, then they might get the fruits as well. But snacks like morning tea or afternoon, it’s usually cakes or breakfast because as they need as well more calories because if they don’t want to eat much….I’ve noticed when it’s kind of like party or birthday or something going on, they offer a slice of watermelon, or slice of strawberry or other berry or any other fruits so you can share…I think it would be nice to have this kind of option during the day… I’ve noticed that the resident may be hungry for fruit, but not with the small pieces, but the bigger pieces where they can see the size of the fruit, like watermelon…so they can grab a slice and enjoy they are really enjoying or apple slices, more this kind of thing during the day to offer the residents…”* Participant 2, Staff member

Additional snacks were often provided in the evening, prior to bedtime to support weight management. Some homes reported that this consists of snacks and drinks such as hot chocolate, horlicks and biscuits.

*“…we give calorically rich drinks such as Horlicks and hot chocolate, and again biscuits which is quite good because if you have those calories just before bed you’re not going to burn them off. So, if you need to maintain weight or not lose any more weight that is an excellent way of doing that.”* Participant 7, Staff member

There was variation in whether residents chose to eat snacks offered at the home or not, with some not wanting sweet snacks but the accessibility of these made it difficult to avoid.

*“Probably have to ask [if wanted something else for snack] but then I don’t … I am not used to eating between meals. So, I don’t, I don’t want it. In fact, I don’t really want cake… but sometimes I fall for it.”* Participant 9, Resident

Commonly, families and friends brought in food items, with residents having snacks in their room such as crisps, chocolates, sweets or fruits. Some participants asked family or friends to brings specific items in, whilst in other cases, family and friends would voluntarily bring in items they thought the resident would like. There was an awareness from staff that chocolates / sweets were being given to residents, as a way of caring for parents and loved ones,

*“Some residents (families) yes, quite a lot, sugary stuff, and sugary drinks and we have to do nothing about this, it is their choice, they want to spoil their parents and they are here and they are over there, it is kind of (a lot)…yeah, I don’t think it’s good, I usually don’t see the fruits on the plate, I see sweets. So, it would be nice to perhaps to talk to the family, but you can take advantages over the residents that lives here…”* Participant 2, Staff member

*“If I wanted treats like Jaffa Cakes or jelly babies, or whatever, they’d bring … they could bring me in whatever I wanted …Or they’d think XXX wants and they would get it [bring it] for me.”* Participant 4, Resident

### Menu development

Catering services differed in each home; catering staff were employed by external companies or by the care home. Menus were reported to be developed either by management at the care home or the head office and changed locally dependent on the preferences of the client group residing in the home. Menus rotated on a 3-4 weekly basis, changed seasonally and were influenced by activities and celebrations. Many care staff reported that residents were involved within the creation of the menu and choosing dishes to include in this menu, along with other care staff. At some homes, this involved having meetings with residents where they could taste the food to help create the food.

*“The chef, the residents, the activities staff, the care staff…and as the management we all have a bit of an input in there… I remember when we first brought the menu, this was taken out to residents meeting, we discussed with them what did they want. They told us what they wanted, and we actually revised the menu…”* Participant 14, Staff member

Staff reported that feedback regarding menu items and food choices are gained from residents. This was undertaken by catering staff speaking with residents after meals or at regular resident meetings.

*“…I will also go around and ask each resident, get to know them, what they like, what they don’t like. although its written down I still go and ask them…but every time at 1 o’clock at lunchtime I go around and ask them did you enjoy that, or didn’t you enjoy that you know just to know what they like, what they didn’t enjoy…”* Participant 6, Staff member

This was confirmed by participating residents who reported that they had the opportunity to provide feedback about the menu choices to the team, for example by talking with chef after a meal. However, it was queried whether the feedback was acted upon.

*“Well one of the chefs come round and talks to us at the table but I am quite vocal in telling them there is too much fried food or too much roasted, or yeah…. I think I am happy I can give them feedback anyway… Whether anyone listens, I don’t know. But I think they do sometimes.”* Participant 9, Resident

### Weight management

Care staff also recognised that there were challenges with residents experiencing appetite loss to ensure that they are eating and getting enough energy. Staff accommodated for this and although provisions were made, they were not always accepted by the resident.

*“…When clients or service users…get to an advance age, sometimes they lose appetite and the challenge that I have, is to see what I could do different to get them to eat a bit more. And then I know we have to base it on the records of what they used to enjoy back then, back in the day, that what you have to offer. Now sometimes it can be a challenge when we prepare the meal, they go the extra mile to do something different and they still don’t have it, that’s kind of a challenge.”* Participant 17, Staff member

The different types of care staff understood their responsibility of raising concerns and how to make a referral if required.

*“… if we’ve spotted something unusual or something going on which we know that shouldn’t be this way, we inform the team leader and the team leader is contacting the GP or organising extra help or support.”* Participant 2, Staff member

The referral process to the dietetics team was reported to be a direct process, however some homes found that there was a long waiting time and found the dieticians to respond quicker to referrals made from the general medical practitioners

*“…the referrals most of the time are made most time by GP, I can make but GP they are more powerful, let’s say, they are more powerful, but usually I need to raise my concern by GP later… I can do a referral straight to the dietician but it is a long to wait….yeah its quicker [to contact the GP]…”* Participant 16, Staff member

### Staff training

Staff reported having had mandatory training which included aspects of oral health and separate modules on nutrition. Additional training was offered varied between homes and was dependent on the role of the staff members. Examples of training included sessions with dieticians (NHS) about fortification of meals or texture modified food training for residents with difficulty swallowing. No one reported about healthy alternatives or sugar reduction and impacts on oral health being included.

*“The training that we have, again, I’m gonna mention e-learning, you know, there’s a unit there that talks about diabetes awareness, now apart from that, there is no other knowledge I get about that [sugar intake] …”* Participant 17, Staff member

In terms of oral health training, some participants reported to have received “in-house” training, but not all could recall having received specific oral health training and in no cases was nutrition integrated within training.

## Discussion

The findings from this study provide valuable insight into the nutrition in care homes, particularly those that are residential in nature and the unique set of challenges which are faced with in this setting.

Having different meal options and flexibility of choices was important, however, there were contradictions between what the residents and care staff participating reported on. At the time of carrying out the interviews, most homes reported that an alternative healthy option was offered at mealtimes, but this wasn’t the case in all homes. Residents reported high volume of unhealthy foods and having to request alternative options. Although they felt comfortable to ask for an alternative if one was not readily presented, respondents highlighted that for those unable to communicate, choice would not be an option. Some residents preferred to go out and pick food, this option is at a personal financial cost and requires a resident to be mobile enough to leave the home, which may not be possible for all. Therefore, there were disparities between residents within homes with the choices available to residents heavily influenced by their ability to articulate their needs, financial freedoms and mobility. This is compounded by the differences in access to food bought in by family and friends. Residents with family and friends visiting regularly and bringing in food had access to a greater range of foods, although in most cases reported, the foods bought in were high sugar snack items, rather than healthy options.

Although approaches to food offers should be standardised within and between care settings, taking a sensible and pragmatic approach is key. Residents have the right to access a wide range of culturally competent and nutritious foods, and central to this is the resident having choices. For some, they will choose to eat food that may not be nutritionally sound, however, the fact that they are in a care setting should not take away their autonomy or choice. Just as people living in the community, care home residents should be offered advice on eating a healthy diet; by offering a range of food options in the home and combining this with food education, the drive should be for the healthy choice to be the easy choice for residents, but it should never be the only choice at the cost diminishing autonomy.

Central to decision making was care home team approaches and attitudes. Care staff were able to build relationships with residents to establish and understand what was usual for the resident in terms of dietary preferences, portion size and food timings, and when to raise concerns with senior care staff, families and healthcare professionals. Importantly, participating staff recognised the care home as the resident’s home, as opposed to a place of work. They reported being sensitive to resident choices and cultural background from a point of admission onwards. Staff were flexible to the care and nutritional needs of the residents to ensure that they were happy and eating. Each care home had some element of flexibility with adapting their menu to their residents’ culture, however some care settings were more fixed than others. This raises the challenge of disparities between homes in their approaches to flexible nutritional choices, which is of particular concern with diverse communities.

In many cases, nutritional choices were often influenced by the need to weight manage the resident, and so food choices were influenced by medical professionals. In this study, we found that many resident choices were based on the willingness of wanting to eat in terms of amount and frequency of meal. Meal supplements given to underweight residents as well as being high in calories, are often high in sugar and are often advised to be consumed between meals. These are often liquid supplements that residents ‘sip’ throughout the day, which significantly increases their dental caries risk. This poses a challenge in integrated ways of working between dietetics and dental teams, with the risk of dental caries rising in those high sugar foods [18]. As a consequence of long waiting times and complex care pathways to dietetics and medical practitioners, carers were often put in a position of making nutritional choices in the interest of the patient, which were often high sugar snacks between meals and before bed. This highlighted the need for comprehensive, topic specific and integrated training, that includes simple suggestions such as intake of liquids through a straw to offer protection of the teeth being given as standard by both dietetic and oral health teams.

Although carers reported independent training on nutrition in different ways, inhouse or online modules, there was no integration of oral health. Oral health and nutrition were reported to be two separate elements of training and not combined to highlight the relationship between the two. With the bidirectional relationship between nutrition and oral health, integration of oral health with wider healthcare is vital and opportunities for all staff to be trained as part of mandatory training must be explored.

Implementation of recommendations from the NICE [25] and CQC [26, 27] reports can help to ensure that care staff have training with specific reference to those residents with challenging behaviour. Formulating an individual oral health care plan for each resident when they are admitted into the care home and establishing records of daily oral care provided by carers in the resident care plans and auditing these on a regular basis to ensure that standards are being maintained in essential. Training regarding oral health and nutrition needs to support staff to reflect the real-life challenges of oral care with residents of varying degrees of cooperation, for example, changes in dysphagia or those on nutritional supplements.

Key stakeholders should work in partnership with care home organisations to integrate consistent, evidence-based oral health and nutrition messaging that can be provided during training for care and kitchen staff. Dietetics team must work in partnership to support care homes in delivering the health choices and change for life agendas by encouraging provision of a range of healthy alternatives at meal and snack times.

There are limitations to the research conducted. There was a small number of participants from four care homes in one London borough which provided initial insight into the challenges faced and they are mainly residential in nature. The research was undertaken prior to the COVID-19 pandemic which had a profound impact on the social care sector. There is limited published evidence of advancement in nutritional and oral health practices since this work took place, however, the generalisability of the findings should be taken with caution. The inclusion criteria included residents who were able to consent for themselves and were English speaking, which will have potentially excluded some residents’ perspectives. However, this was acknowledged by participants themselves. Participating residents reported in their interviews that they were aware that they had more independence and freedom than other residents. This was also the case for residents who may not have family members visiting. Participants reported being confident to advocate their choices and request or obtain food or drink items they desired, which would not have been the case for all. In future studies, broadening the inclusion criteria, and speaking to residents’ family members would provide further insights. This would enable a fuller understanding of the issues at hand and if nutritional choices are being honoured, particularly for those residents unable to advocate for themselves. Secondly, input from the nutrition/dietetics team would have been useful to understand the wider system and how they have influence in care homes. Further research should consider capturing views from wider multidisciplinary teams involved in both nutrition and oral health and explore the balance between resident autonomy and evidence informed care.

## Conclusion

This study provides insights into the nutritional routines of care homes, the views of residents on the choices available to them and how these relate to oral health. There are clear disparities around nutritional practises between care home teams and between residents residing in the same home. Care homes should ensure that resident centred care is provided for all residents, being mindful of their personal circumstances, willingness and ability to participate. A range of healthy food options should ideally be offered as a standard with suitable alternate options being accessible at every meal. The oral health implications of peoples prescribed and available nutritional practices in care homes requires further research. Nevertheless, increasing the choice of healthy option available would be a step on the journey to create a supportive environment that helps to make the healthy choice an easier choice.

The complexities of the health and social care landscape result in little consistency of integration across the system. The challenges of the social care landscape have been perpetuated and highlighted because of the COVID-19 pandemic [29] and Brexit [30, 31]. Staff retention and turnover, the vulnerability of residents and staff and chronic lack of funding has resulted in a highly pressurised environment and system. Policy and support are needed to enable collaborative action across health and social care, including dental systems [32]. This at its core should be shaped by care home teams, residents and family members experiences to ensure a ground up, patient centred approach to planning and care delivery.

## Data Availability

The datasets used and/or analysed during the current study are available from the corresponding author on reasonable request.

## References

[1] World Health Organization. Ageing and Health: World Health Organization. Available at: https://www.who.int/news-room/fact-sheets/detail/ageing-and-health.2018

[2] Office for National Statistics. Health state life expectancies in England, Northern Ireland and Wales: between 2011 to 2013 and 2020 to 2022. March. Available at: Health state life expectancies in England, Northern Ireland and Wales - Office for National Statistics (ons.gov.uk) 2024.

[3] Carehome.co.uk. Care home stats: number of settings, population & workforce. April. Care home stats: number of settings, population & workforce - carehome.co.uk advice 2024.

[4] Beaven, A, Marshman, Z Barriers and facilitators to accessing oral healthcare for older people in the UK: a scoping review. Br Dent J. 10.1038/s41415-024-7740-x 2024. 10.1038/s41415-024-7740-x 39152268

[5] Patel R, Mian M, Robertson C, Pitts NB, Gallagher JE. Crisis in care homes: the dentists don’t come. BDJ Open. 2021;7:20. 34103478 10.1038/s41405-021-00075-4 PMC8186358

[6] Borreani E, Wright D, Scambler S, Gallagher JE. Minimising barriers to dental care in older people. BMC Oral health. 2008;8:7. 18366785 10.1186/1472-6831-8-7 PMC2335092

[7] Borreani E, Jones K, Wright D, Scambler S, Gallagher JE. Improving access to dental care for older people. Dent Update. 2010;37:297–8. 20669708 10.12968/denu.2010.37.5.297

[8] Borreani E, Jones K, Scambler S, Gallagher JE. Informing the debate on oral health care for older people: a qualitative study of older people’s views on oral health and oral health care. Gerodontology. 2010;27:11–8. 19817878 10.1111/j.1741-2358.2009.00274.x

[9] Lockhart PB, Brennan MT, Thornhill M, Michalowicz BS, Noll J, Bahrani-Mougeot FK, et al. Poor oral hygiene as a risk factor for infective endocarditis-related bacteremia. J Am Dent Assoc. 2009;140:1238–44. 19797553 10.14219/jada.archive.2009.0046 PMC2770162

[10] Ziebolz D, Werner C, Schmalz G, Nitschke I, Haak R, Mausberg RF, et al. Oral Health and nutritional status in nursing home residents—results of an explorative cross-sectional pilot study. BMC Geriatr. 2017;17:39. 28143415 10.1186/s12877-017-0429-0 PMC5282867

[11] Saunders MJ, Stattmiller SP, Kirk KM. Oral health issues in the nutrition of institutionalized elders. J Nutr Elder. 2007;26:39–58. 18285292 10.1300/j052v26n03_03

[12] Walls AW, Steele JG, Sheiham A, Marcenes W, Moynihan PJ. Oral health and nutrition in older people. J Public Health Dent. 2000;60:304–7. 11243051 10.1111/j.1752-7325.2000.tb03339.x

[13] Locker D, Matear D, Stephens M, Jokovic A. Oral health-related quality of life of a population of medically compromised elderly people. Community Dent Health. 2002;19:90–7. 12146588

[14] Chan AKY, Tsang YC, Jiang CM, Leung KCM, Lo ECM, Chu CH. Diet, nutrition, and oral health in older adults: a review of the literature. Dent J (Basel). 2023;11:222 37754342 10.3390/dj11090222 PMC10528506

[15] Watson S, McGowan L, McCrum LA, Cardwell CR, McGuinness B, Moore C, et al. The impact of dental status on perceived ability to eat certain foods and nutrient intakes in older adults: cross-sectional analysis of the UK National Diet and Nutrition Survey 2008–2014. Int J Behav Nutr Phys Act. 2019;16:43. 31088468 10.1186/s12966-019-0803-8 PMC6518671

[16] Walls AWG, Steele JG. The relationship between oral health and nutrition in older people. Mechanisms Ageing Dev. 2004;125:853–7. 10.1016/j.mad.2004.07.011 15563930

[17] British Dietetics Association. Spotting and treating malnutrition. July. Available at: https://www.bda.uk.com/resource/malnutrition.html 2022.

[18] GOV.UK. Delivering better oral health: an evidence-based toolkit for prevention. Updated. Available at: Delivering better oral health: an evidence-based toolkit for prevention - GOV.UK (www.gov.uk) 2024. 10.12968/denu.2008.35.7.460 18853715

[19] Kassebaum NJ, Smith AGC, Bernabé E, Fleming TD, Reynolds AE, Vos T, et al. Global, Regional, and National Prevalence, Incidence, and Disability-Adjusted Life Years for Oral Conditions for 195 Countries, 1990–2015: A Systematic Analysis for the Global Burden of Diseases, Injuries, and Risk Factors. J Dent Res. 2017;96:380–7. 28792274 10.1177/0022034517693566 PMC5912207

[20] NHS England Digital. Adult Dental Health Survey 2009 – Summary report and thematic series. March. Available at: https://digital.nhs.uk/data-and-information/publications/statistical/adult-dental-health-survey/adult-dental-health-survey-2009-summary-report-and-thematic-series 2011.

[21] Moynihan P. Sugars and dental caries: evidence for setting a recommended threshold for intake. Adv Nutr. 2016;7:149–56. 10.3945/an.115.009365. 26773022 10.3945/an.115.009365 PMC4717883

[22] Moore D, Davies GM. A summary of knowledge about the oral health of older people in England and Wales. Community Dent Health. 2016;33:262–6. 28537362 10.1922/CDH_3884Moore05

[23] Karki AJ, Monaghan N, Morgan M. Oral health status of older people living in care homes in Wales. Br Dent J 2015;219:331–4. 26450249 10.1038/sj.bdj.2015.756

[24] Tomson M, Watson F, Taylor-Weetman K, Morris AJ, Wilson KI. West Midlands Care Home Dental Survey 2011: part 2. Results of clinical survey of care home residents. Br Dent J. 2015;219:349–53. 26450252 10.1038/sj.bdj.2015.758

[25] National Institute for Health and Care Excellence (NICE). Oral Health for adults in care homes- NICE guideline [NG48]. July 2016. Available at: Overview | Oral health for adults in care homes | Guidance | NICE.

[26] Care Quality Commission (CQC). Smiling matters: oral health care in care homes. June 2019. Available at: Smiling matters: oral health care in care homes - Care Quality Commission (cqc.org.uk).

[27] Care Quality Commission (CQC). Smiling matters: Oral health care in care homes – progress report. March 2023. Available at: Smiling matters: Oral health in care homes - progress report - Care Quality Commission (cqc.org.uk).

[28] Ritchie J, Lewis J. Qualitative research practice: a guide for social science students and researchers. Second edition. ed. SAGE Publications, 2003.

[29] Owens Janine, Young Alys, Allen Rosie, Pearson Amelia, Cartney Patricia, Robinson Catherine, et al. The impact of COVID-19 on social care and social work in the UK: a scoping review. Br J Soc Work. 2024;54:885–904.

[30] Turnpenny A, Hussein S. Migrant home care workers in the UK: a scoping review of outcomes and sustainability and implications in the context of Brexit. Int Migr Integr. 2022;23:23–42. 10.1007/s12134-021-00807-3 PMC8007051 33814989

[31] McCarey M, Dayan M, Jarman H, Hervey T, Fahy N, Bristow D, et al. *Health and Brexit: Six Years On*. London, England: Nuffield Trust. Print. 2022

[32] Patel R, Gallagher JE. Healthy ageing and oral health: priority, policy and public health. BDJ Open. 2024;10:79. 39379352 10.1038/s41405-024-00262-z PMC11461822

